# Supplementary material for: Loop-Mediated Isothermal Amplification Allows Rapid, Simple and Accurate Molecular Diagnosis of Human Cutaneous and Visceral Leishmaniasis Caused by Leishmania infantum When Compared to PCR
Source: Microorganisms. 2021 Mar 16;9(3):610. doi: 10.3390/microorganisms9030610 (PMC7999953; doi:10.3390/microorganisms9030610)
Supplement: Supplementary file 1 [file microorganisms-09-00610-s001.zip › microorganisms-1143587-supplementary.pptx]

## Slide 1
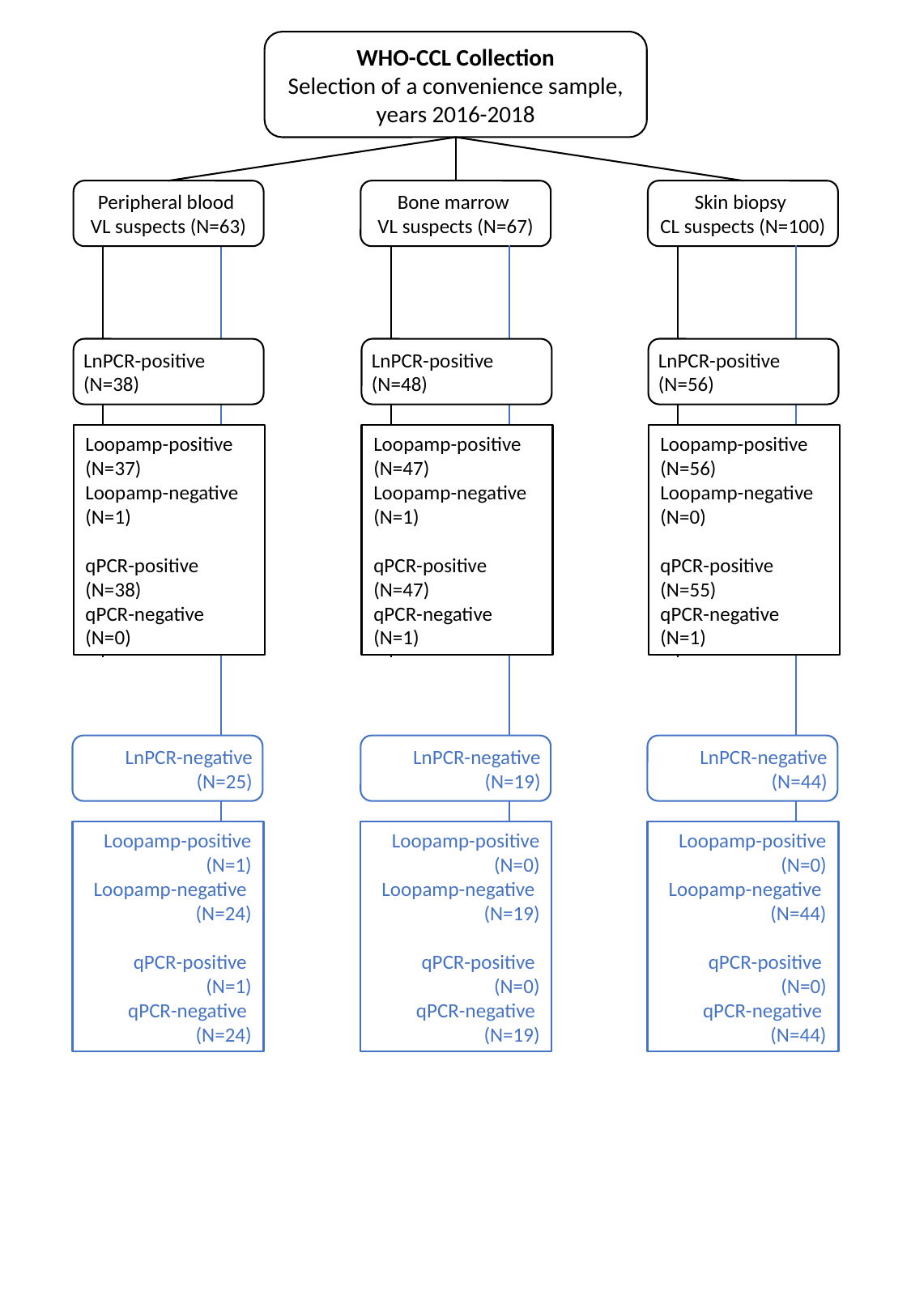

WHO-CCL Collection
Selection of a convenience sample, years 2016-2018
Peripheral blood
VL suspects (N=63)
Bone marrow
VL suspects (N=67)
Skin biopsy
CL suspects (N=100)
LnPCR-positive (N=38)
LnPCR-positive (N=48)
LnPCR-positive (N=56)
Loopamp-positive (N=37)
Loopamp-negative (N=1)
qPCR-positive (N=38)
qPCR-negative (N=0)
Loopamp-positive (N=47)
Loopamp-negative (N=1)
qPCR-positive (N=47)
qPCR-negative (N=1)
Loopamp-positive (N=56)
Loopamp-negative (N=0)
qPCR-positive (N=55)
qPCR-negative (N=1)
LnPCR-negative (N=25)
LnPCR-negative (N=19)
LnPCR-negative (N=44)
Loopamp-positive (N=1)
Loopamp-negative (N=24)
qPCR-positive
(N=1)
qPCR-negative
(N=24)
Loopamp-positive (N=0)
Loopamp-negative (N=19)
qPCR-positive
(N=0)
qPCR-negative
(N=19)
Loopamp-positive (N=0)
Loopamp-negative (N=44)
qPCR-positive
(N=0)
qPCR-negative
(N=44)
